# The genome sequence of the red compost earthworm, *Lumbricus rubellus *(Hoffmeister, 1843)

**DOI:** 10.12688/wellcomeopenres.19834.1

**Published:** 2023-08-18

**Authors:** Stephen Short, Amaia Green Etxabe, Alex Robinson, David Spurgeon, Peter Kille, Wellcome Sanger Institute Tree of Life programme, Wellcome Sanger Institute Scientific Operations: DNA Pipelines collective, Tree of Life Core Informatics collective, Darwin Tree of Life Consortium

**Affiliations:** 1 UK Centre for Ecology & Hydrology, Wallingford, England, UK; 2 Cardiff University, Cardiff, Wales, UK

**Keywords:** Lumbricus rubellus, red compost earthworm, genome sequence, chromosomal, Haplotaxida

## Abstract

We present a genome assembly from an individual *Lumbricus rubellus* (the red compost earthworm; Annelida; Clitellata; Haplotaxida; Lumbricidae). The genome sequence is 787.5 megabases in span. Most of the assembly is scaffolded into 18 chromosomal pseudomolecules. The mitochondrial genome has also been assembled and is 15.81 kilobases in length. Gene annotation of this assembly on Ensembl identified 33,426 protein coding genes.

## Species taxonomy

Eukaryota; Metazoa; Eumetazoa; Bilateria; Protostomia; Spiralia; Lophotrochozoa; Annelida; Clitellata; Oligochaeta; Crassiclitellata; Lumbricina; Lumbricidae; Lumbricinae; *Lumbricus*; *Lumbricus rubellus* complex (Hoffmeister, 1843) (NCBI:txid35632).

## Background

**Figure 1.**
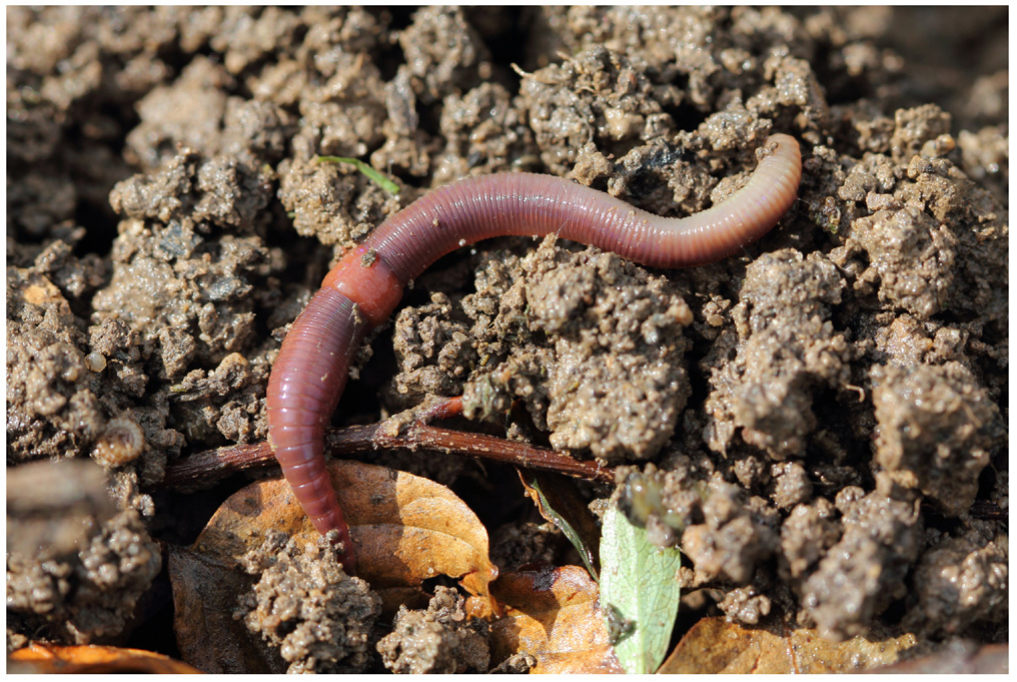
Photograph of *Lumbricus rubellus* by Holger Casselmann (CC-BY-SA 3.0).

*Lumbricus rubellus* (Hoffmeister, 1843) is an earthworm that feeds on decaying organic matter near the soil surface. Up to 130 mm in length, it has a cylindrical body in cross section except for a flattened posterior, possessing a purplish pigmentation dorsally at the head-end ( James, 2010) ( Figure 1). Though native to Europe, *L. rubellus* has become an invasive species through accidental and deliberate transport worldwide ( James, 2010; Klein *et al.*, 2020).

Typical of lumbricid species, *L. rubellus* exhibits highly divergent mitochondrial lineages, with evidence of five distinct lineages across Europe ( Giska *et al.*, 2015). However, in the UK, just two (lineages A and B) are found, a reduced diversity likely due to limited re-colonisation after the loss of the land bridge to continental Europe following glacial retreat ( Jones *et al.*, 2016). Despite this divergence, the mitochondrial lineages are not entirely reproductively isolated ( Giska *et al.*, 2015), even though reproductive pheromone variation and different habitat preferences reinforce lineage separation ( Jones *et al.*, 2016; Spurgeon *et al.*, 2016). The genome presented here represents the A lineage, which appears to be the more dominant of the two lineages in the UK ( Spurgeon *et al.*, 2016).

Renowned ecologist John Stewart Collis described earthworms as “Eyeless, legless, faceless, voiceless, the earth-worm is not much to look at – a mere squirming piece of flesh,” yet capable of “remarkable works”. The genome of *L. rubellus*, a litter-inhabiting and cosmopolitan species, will provide insights into their particular abilities, including their unique metabolism, potential for tissue regeneration, as well as their capacity to colonise soils with varying contamination profiles and dramatic proton concentration differences. The relevance of *L. rubellus* to ecotoxicology ( Morgan *et al.*, 2004), ecology ( Uvarov, 2009), biotechnology ( Bakar *et al.*, 2011), and evolutionary biology ( Ferrier, 2012) makes this genome a vital resource for a broad range of scientific disciplines.

We present the complete genome sequence of *Lumbricus rubellus*, an earthworm species collected from Dinas Powys in south Wales as part of the Darwin Tree of Life Project. This project is a collaborative effort to sequence all named eukaryotic species in the Atlantic Archipelago of Britain and Ireland.

## Genome sequence report

The genome was sequenced from one *Lumbricus rubellus* from a culture collection held at the Kille Lab, University of Cardiff. A total of 30-fold coverage in Pacific Biosciences single-molecule HiFi long reads and 36-fold coverage in 10X Genomics read clouds were generated. Primary assembly contigs were scaffolded with chromosome conformation Hi-C data. Manual assembly curation corrected 459 missing joins or misjoins and removed 358 haplotypic duplications, reducing the assembly length by 14.03% and the scaffold number by 51.72%, and increasing the scaffold N50 by 0.15%.

**Figure 2.**
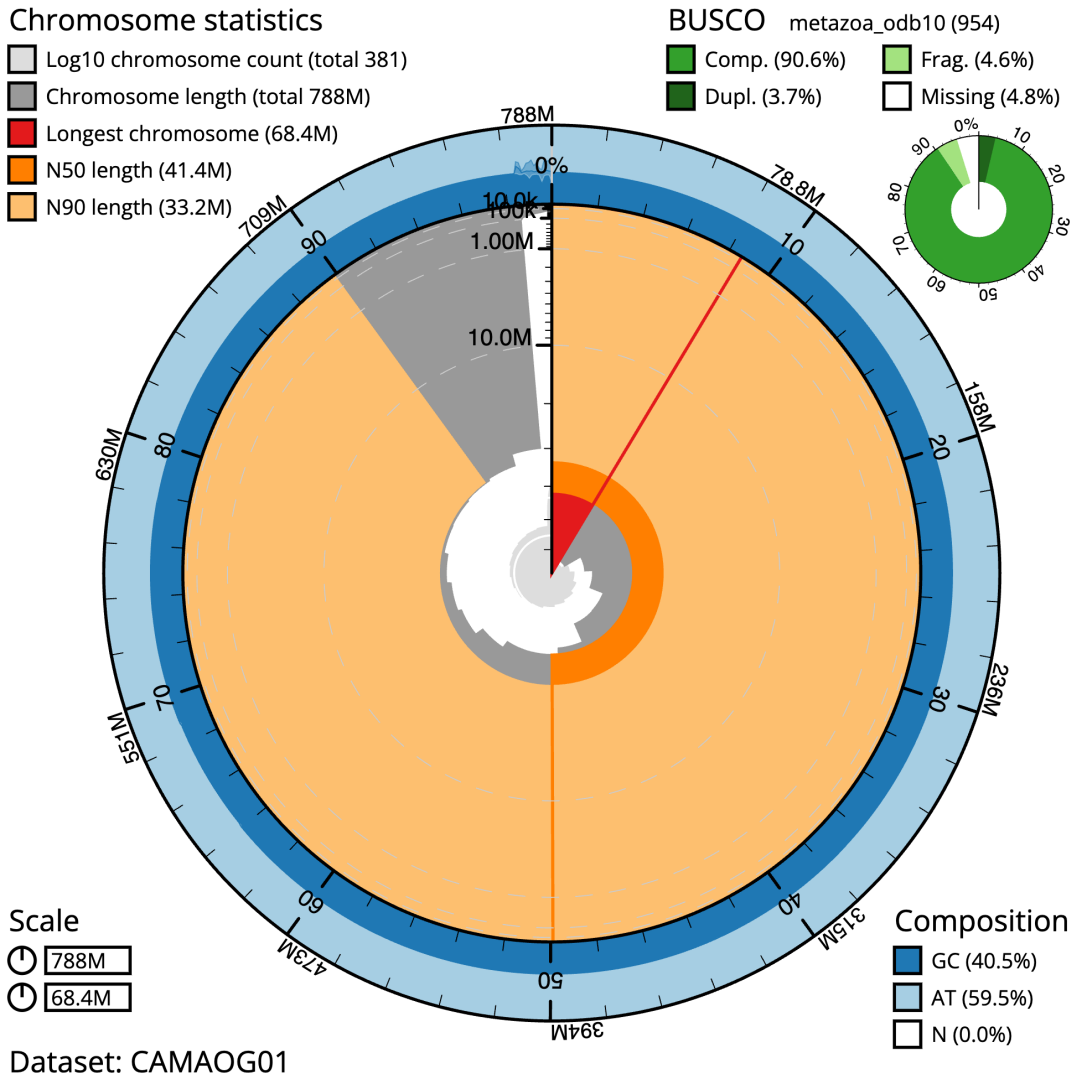
Genome assembly of *Lumbricus rubellus*, wcLumRube1.1: metrics. The BlobToolKit Snailplot shows N50 metrics and BUSCO gene completeness. The main plot is divided into 1,000 size-ordered bins around the circumference with each bin representing 0.1% of the 787,530,981 bp assembly. The distribution of scaffold lengths is shown in dark grey with the plot radius scaled to the longest scaffold present in the assembly (68,399,915 bp, shown in red). Orange and pale-orange arcs show the N50 and N90 scaffold lengths (41,365,854 and 33,209,857 bp), respectively. The pale grey spiral shows the cumulative scaffold count on a log scale with white scale lines showing successive orders of magnitude. The blue and pale-blue area around the outside of the plot shows the distribution of GC, AT and N percentages in the same bins as the inner plot. A summary of complete, fragmented, duplicated and missing BUSCO genes in the metazoa_odb10 set is shown in the top right. An interactive version of this figure is available at https://blobtoolkit.genomehubs.org/view/Lumbricus%20rubellus/dataset/CAMAOG01/snail.

The final assembly has a total length of 787.5 Mb in 380 sequence scaffolds with a scaffold N50 of 41.4 Mb ( Table 1). Most (98.53%) of the assembly sequence was assigned to 18 chromosomal-level scaffolds. Chromosome-scale scaffolds confirmed by the Hi-C data are named in order of size ( Figure 2– Figure 5; Table 2). While not fully phased, the assembly deposited is of one haplotype. Contigs corresponding to the second haplotype have also been deposited. The mitochondrial genome was also assembled and can be found as a contig within the multifasta file of the genome submission.

**Table 1.** Genome data for *Lumbricus rubellus*, wcLumRube1.1.

Project accession data		
Assembly identifier	wcLumRube1.1	
Species	*Lumbricus rubellus*	
Specimen	wcLumRube1	
NCBI taxonomy ID	35632	
BioProject	PRJEB53406	
BioSample ID	SAMEA7524021	
Isolate information	wcLumRube1: body wall tissue (DNA sequencing) wcLumRube5: body wall tissue (Hi-C scaffolding) wcLumRube2: body wall tissue (RNA sequencing)	
Assembly metrics *		Benchmark
Consensus quality (QV)	52.2	*≥ 50*
*k*-mer completeness	99.98%	*≥ 95%*
BUSCO **	C:90.6%[S:86.9%,D:3.7%], F:4.6%,M:4.8%,n:954	*C ≥ 95%*
Percentage of assembly mapped to chromosomes	98.53%	*≥ 95%*
Sex chromosomes	-	*localised homologous pairs*
Organelles	Mitochondrial genome assembled	*complete single alleles*
Raw data accessions		
PacificBiosciences SEQUEL IIe	ERR9836431	
10X Genomics Illumina	ERR9837131, ERR9837132, ERR9837133, ERR9837134	
Hi-C Illumina	ERR9837135, ERR9837137, ERR9837138	
RNA-Seq	ERR9837136	
Genome assembly		
Assembly accession	GCA_945859605.1	
*Accession of alternate haplotype*	GCA_945859625.1	
Span (Mb)	787.5	
Number of contigs	2261	
Contig N50 length (Mb)	0.7	
Number of scaffolds	380	
Scaffold N50 length (Mb)	41.4	
Longest scaffold (Mb)	68.4	
Genome annotation		
Number of protein-coding genes	33,426	
Number of non-coding genes	13,823	
Number of gene transcripts	69,438	

* Assembly metric benchmarks are adapted from column VGP-2020 of “Table 1: Proposed standards and metrics for defining genome assembly quality” from ( Rhie *et al.*, 2021). ** BUSCO scores based on the metazoa_odb10 BUSCO set using v5.3.2. C = complete [S = single copy, D = duplicated], F = fragmented, M = missing, n = number of orthologues in comparison. A full set of BUSCO scores is available at https://blobtoolkit.genomehubs.org/view/Lumbricus%20rubellus/dataset/CAMAOG01/busco.

**Figure 3.**
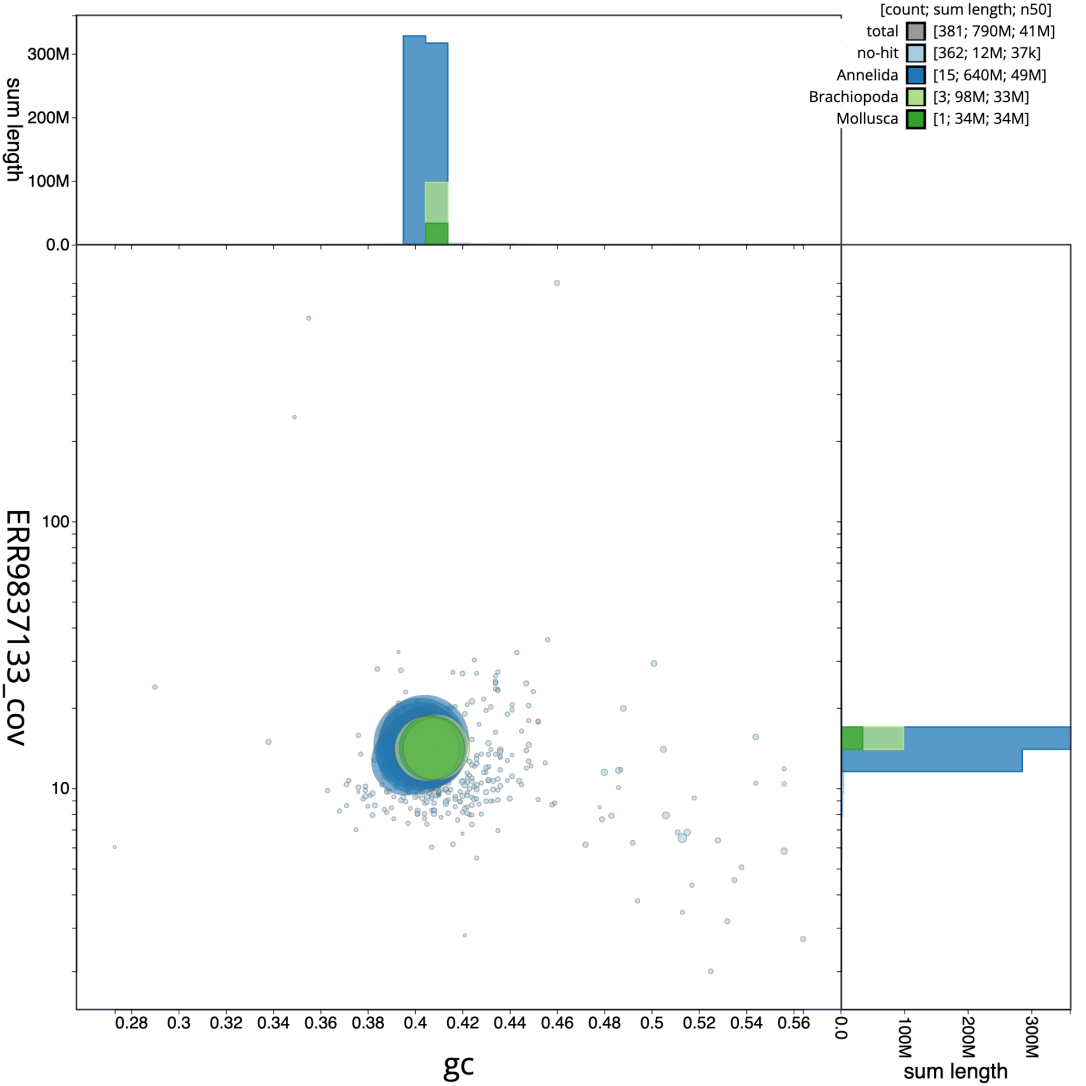
Genome assembly of *Lumbricus rubellus*, wcLumRube1.1: BlobToolKit GC-coverage plot. Scaffolds are coloured by phylum. Circles are sized in proportion to scaffold length. Histograms show the distribution of scaffold length sum along each axis. An interactive version of this figure is available at https://blobtoolkit.genomehubs.org/view/Lumbricus%20rubellus/dataset/CAMAOG01/blob.

**Figure 4.**
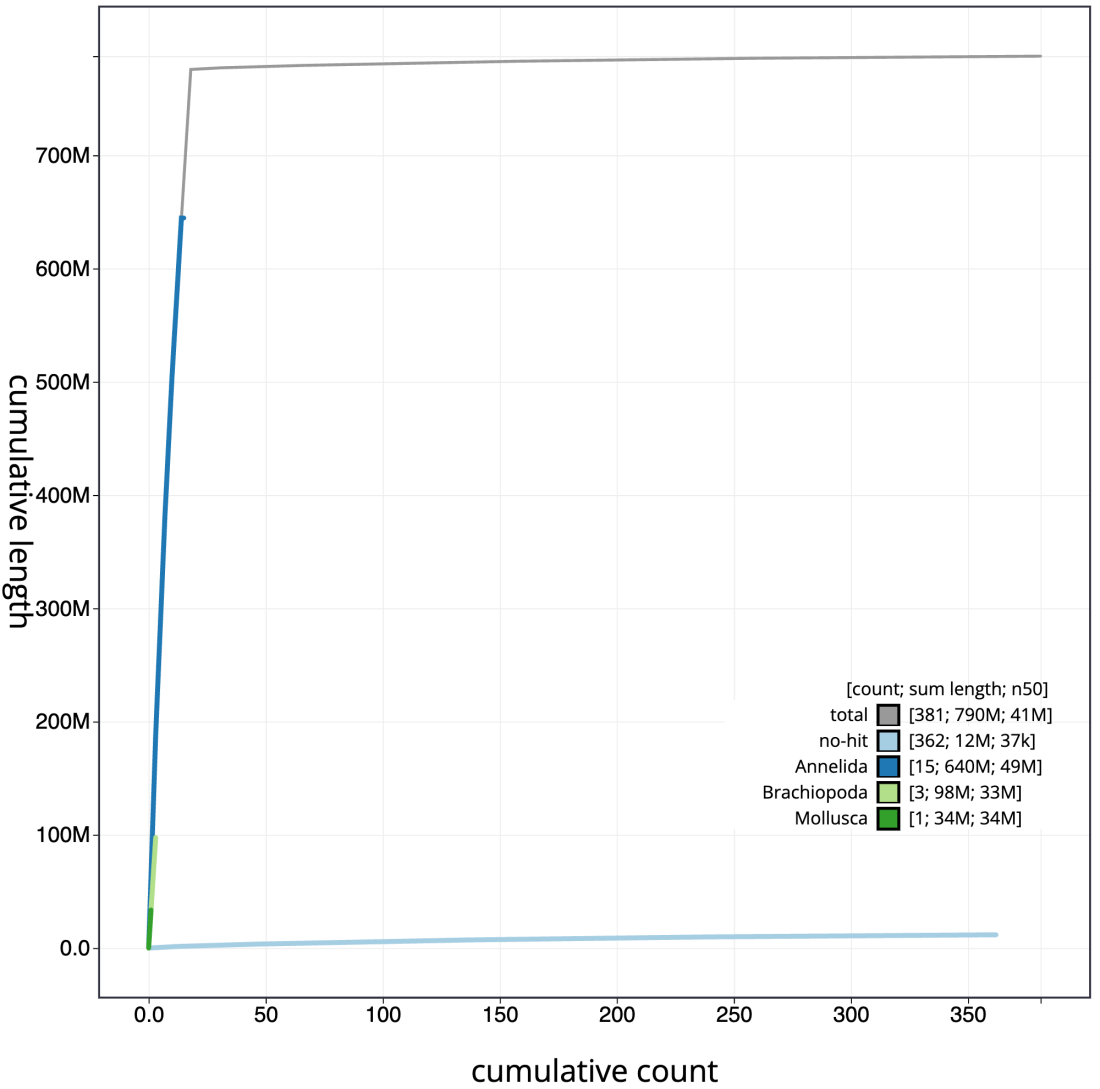
Genome assembly of *Lumbricus rubellus*, wcLumRube1.1: BlobToolKit cumulative sequence plot. The grey line shows cumulative length for all scaffolds. Coloured lines show cumulative lengths of scaffolds assigned to each phylum using the buscogenes taxrule. An interactive version of this figure is available at https://blobtoolkit.genomehubs.org/view/Lumbricus%20rubellus/dataset/CAMAOG01/cumulative.

**Figure 5.**
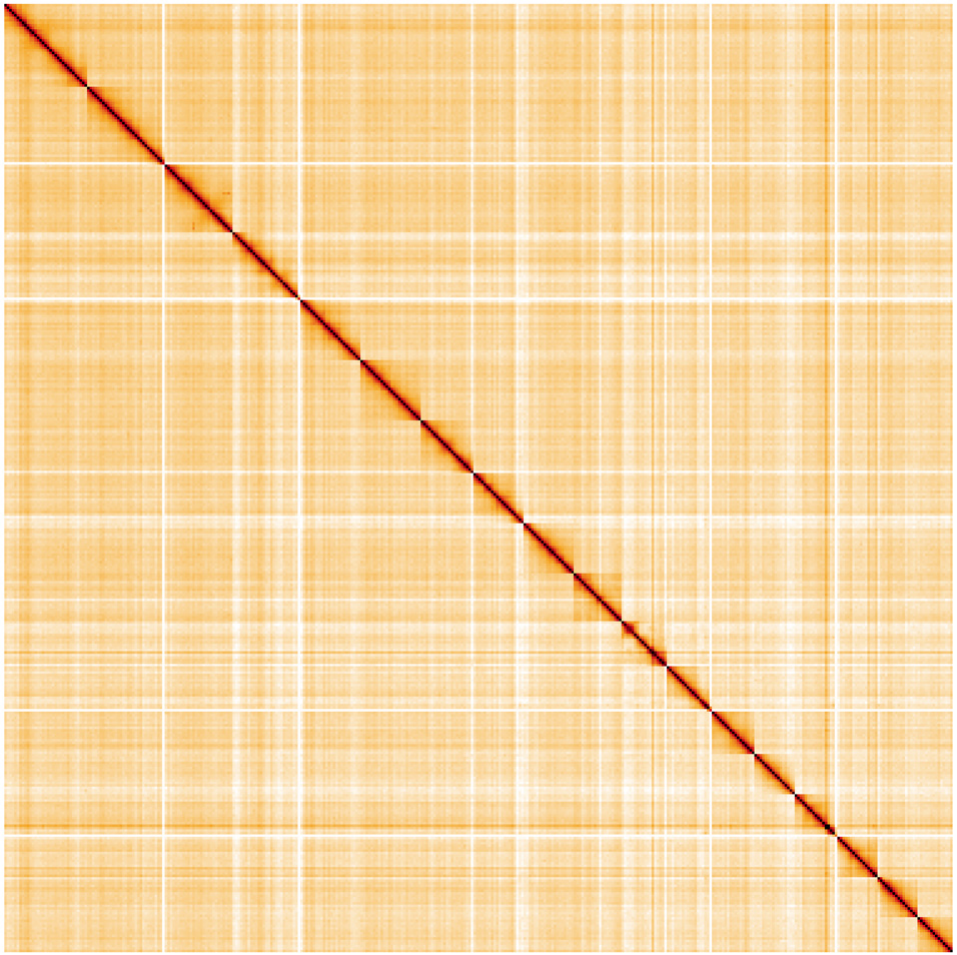
Genome assembly of *Lumbricus rubellus*, wcLumRube1.1: Hi-C contact map of the wcLumRube1.1 assembly, visualised using HiGlass. Chromosomes are shown in order of size from left to right and top to bottom. An interactive version of this figure may be viewed at https://genome-note-higlass.tol.sanger.ac.uk/l/?d=O4FKa6EsSKmCY1sc7Gjldw.

**Table 2.** Chromosomal pseudomolecules in the genome assembly of *Lumbricus rubellus*, wcLumRube1.

INSDC accession	Chromosome	Length (Mb)	GC%
OX243811.1	1	68.4	40.0
OX243812.1	2	62.94	40.5
OX243813.1	3	56.54	40.0
OX243814.1	4	53.92	40.0
OX243815.1	5	49.32	40.5
OX243816.1	6	49.16	40.5
OX243817.1	7	43.19	40.5
OX243818.1	8	41.37	40.5
OX243819.1	9	41.33	40.5
OX243820.1	10	39.25	40.5
OX243821.1	11	36.88	39.5
OX243822.1	12	35.89	40.5
OX243823.1	13	34.73	41.0
OX243824.1	14	33.68	40.5
OX243825.1	15	33.54	40.5
OX243826.1	16	33.21	40.5
OX243827.1	17	32.7	40.5
OX243828.1	18	29.72	41.0
OX243829.1	MT	0.02	35.5

The estimated Quality Value (QV) of the final assembly is 52.2 with *k*-mer completeness of 99.98%, and the assembly has a BUSCO v5.3.2 completeness of 90.6% (single = 86.9%, duplicated = 3.7%), using the metazoa_odb10 reference set ( *n* = 954).

Metadata for specimens, spectral estimates, sequencing runs, contaminants and pre-curation assembly statistics can be found on the Wellcome Sanger Tree of Life website.

## Genome annotation report

The *Lumbricus rubellus* genome assembly (GCA_945859605.1) was annotated using the Ensembl rapid annotation pipeline ( Table 1; https://rapid.ensembl.org/Lumbricus_rubellus__GCA_945859605.1/Info/Index). The resulting annotation includes 69,438 transcribed mRNAs from 33,426 protein-coding and 13,823 non-coding genes.

## Methods

### Sample acquisition and nucleic acid extraction

The *Lumbricus rubellus* specimens used for genome sequencing (specimen ID SAN0001201, individual wcLumRube1), Hi-C scaffolding (specimen ID SAN0001205, wcLumRube5) and RNA sequencing (specimen ID SAN0001202, wcLumRube2) were obtained from a culture collection held at UKCEH, Wallingford, United Kingdom on 2020-03-17. This culture was generated (and regularly supplemented) using *L. rubellus* collected from Dinas Powys, Wales, United Kingdom (51.44, –3.24). The specimens were collected by Stephen Short, Amaia Green Etxabe and Alex Robinson (UK Centre for Ecology and Hydrology). The specimens were identified by Stephen Short and then flash frozen in liquid nitrogen.

DNA was extracted at the Tree of Life laboratory, Wellcome Sanger Institute (WSI). The wcLumRube1 sample was weighed and dissected on dry ice with tissue set aside for Hi-C sequencing. Bodywall tissue was disrupted using a Nippi Powermasher fitted with a BioMasher pestle. High molecular weight (HMW) DNA was extracted using the Qiagen MagAttract HMW DNA extraction kit. Low molecular weight DNA was removed from a 20-ng aliquot of extracted DNA using the 0.8X AMpure XP purification kit prior to 10X Chromium sequencing; a minimum of 50 ng DNA was submitted for 10X sequencing. HMW DNA was sheared into an average fragment size of 12–20 kb in a Megaruptor 3 system with speed setting 30. Sheared DNA was purified by solid-phase reversible immobilisation using AMPure PB beads with a 1.8X ratio of beads to sample to remove the shorter fragments and concentrate the DNA sample. The concentration of the sheared and purified DNA was assessed using a Nanodrop spectrophotometer and Qubit Fluorometer and Qubit dsDNA High Sensitivity Assay kit. Fragment size distribution was evaluated by running the sample on the FemtoPulse system.

RNA was extracted from body wall tissue of wcLumRube2 in the Tree of Life Laboratory at the WSI using TRIzol, according to the manufacturer’s instructions. RNA was then eluted in 50 μl RNAse-free water and its concentration assessed using a Nanodrop spectrophotometer and Qubit Fluorometer using the Qubit RNA Broad-Range (BR) Assay kit. Analysis of the integrity of the RNA was done using Agilent RNA 6000 Pico Kit and Eukaryotic Total RNA assay.

### Sequencing

Pacific Biosciences HiFi circular consensus and 10X Genomics read cloud DNA sequencing libraries were constructed according to the manufacturers’ instructions. Poly(A) RNA-Seq libraries were constructed using the NEB Ultra II RNA Library Prep kit. DNA and RNA sequencing was performed by the Scientific Operations core at the WSI on Pacific Biosciences SEQUEL II (HiFi), Illumina HiSeq 4000 (RNA-Seq) and HiSeq X Ten (10X) instruments. Hi-C data were also generated from body wall tissue of wcLumRube1 and wcLumRube5 using the Arima2 kit and sequenced on the Illumina NovaSeq 6000 and HiSeq X Ten instruments.

### Genome assembly, curation and evaluation

Assembly was carried out with Hifiasm ( Cheng *et al.*, 2021) and haplotypic duplication was identified and removed with purge_dups ( Guan *et al.*, 2020). One round of polishing was performed by aligning 10X Genomics read data to the assembly with Long Ranger ALIGN, calling variants with FreeBayes ( Garrison & Marth, 2012). The assembly was then scaffolded with Hi-C data ( Rao *et al.*, 2014) using YaHS ( Zhou *et al.*, 2023). The assembly was checked for contamination and corrected as described previously ( Howe *et al.*, 2021). Manual curation was performed using HiGlass ( Kerpedjiev *et al.*, 2018) and Pretext ( Harry, 2022). The mitochondrial genome was assembled using MitoHiFi ( Uliano-Silva *et al.*, 2023), which runs MitoFinder ( Allio *et al.*, 2020) or MITOS ( Bernt *et al.*, 2013) and uses these annotations to select the final mitochondrial contig and to ensure the general quality of the sequence.

A Hi-C map for the final assembly was produced using bwa-mem2 ( Vasimuddin *et al.*, 2019) in the Cooler file format ( Abdennur & Mirny, 2020). To assess the assembly metrics, the *k*-mer completeness and QV consensus quality values were calculated in Merqury ( Rhie *et al.*, 2020). This work was done using Nextflow ( Di Tommaso *et al.*, 2017) DSL2 pipelines “sanger-tol/readmapping” ( Surana *et al.*, 2023a) and “sanger-tol/genomenote” ( Surana *et al.*, 2023b). The genome was analysed within the BlobToolKit environment ( Challis *et al.*, 2020) and BUSCO scores ( Manni *et al.*, 2021; Simão *et al.*, 2015) were calculated.

Table 3 contains a list of relevant software tool versions and sources.

**Table 3.** Software tools: versions and sources.

Software tool	Version	Source
BlobToolKit	3.4.0	https://github.com/blobtoolkit/blobtoolkit
BUSCO	5.3.2	https://gitlab.com/ezlab/busco
FreeBayes	1.3.1-17-gaa2ace8	https://github.com/freebayes/freebayes
Hifiasm	0.16.1-r375	https://github.com/chhylp123/hifiasm
HiGlass	1.11.6	https://github.com/higlass/higlass
Long Ranger ALIGN	2.2.2	https://support.10xgenomics.com/genome-exome/ software/pipelines/latest/advanced/other-pipelines
Merqury	MerquryFK	https://github.com/thegenemyers/MERQURY.FK
MitoHiFi	2	https://github.com/marcelauliano/MitoHiFi
PretextView	0.2	https://github.com/wtsi-hpag/PretextView
purge_dups	1.2.3	https://github.com/dfguan/purge_dups
sanger-tol/genomenote	v1.0	https://github.com/sanger-tol/genomenote
sanger-tol/readmapping	1.1.0	https://github.com/sanger-tol/readmapping/tree/1.1.0
YaHS	yahs-1.1.91eebc2	https://github.com/c-zhou/yahs

### Genome annotation

The Ensembl gene annotation system ( Aken *et al.*, 2016) was used to generate annotation for the *Lumbricus rubellus* assembly (GCA_945859605.1). Annotation was created primarily through alignment of transcriptomic data to the genome, with gap filling via protein-to-genome alignments of a select set of proteins from UniProt ( UniProt Consortium, 2019).

### Wellcome Sanger Institute – Legal and Governance

The materials that have contributed to this genome note have been supplied by a Darwin Tree of Life Partner. The submission of materials by a Darwin Tree of Life Partner is subject to the **‘Darwin Tree of Life Project Sampling Code of Practice’**, which can be found in full on the Darwin Tree of Life website here. By agreeing with and signing up to the Sampling Code of Practice, the Darwin Tree of Life Partner agrees they will meet the legal and ethical requirements and standards set out within this document in respect of all samples acquired for, and supplied to, the Darwin Tree of Life Project.

Further, the Wellcome Sanger Institute employs a process whereby due diligence is carried out proportionate to the nature of the materials themselves, and the circumstances under which they have been/are to be collected and provided for use. The purpose of this is to address and mitigate any potential legal and/or ethical implications of receipt and use of the materials as part of the research project, and to ensure that in doing so we align with best practice wherever possible. The overarching areas of consideration are:

•     Ethical review of provenance and sourcing of the material

•     Legality of collection, transfer and use (national and international)

Each transfer of samples is further undertaken according to a Research Collaboration Agreement or Material Transfer Agreement entered into by the Darwin Tree of Life Partner, Genome Research Limited (operating as the Wellcome Sanger Institute), and in some circumstances other Darwin Tree of Life collaborators.

## Data Availability

European Nucleotide Archive: *Lumbricus rubellus* (red compost earthworm). Accession number PRJEB53406; https://identifiers.org/ena.embl/PRJEB53406. ( Wellcome Sanger Institute, 2022) The genome sequence is released openly for reuse. The *Lumbricus rubellus* genome sequencing initiative is part of the Darwin Tree of Life (DToL) project. All raw sequence data and the assembly have been deposited in INSDC databases. Raw data and assembly accession identifiers are reported in Table 1. Members of the Wellcome Sanger Institute Tree of Life programme are listed here: https://doi.org/10.5281/zenodo.4783585. Members of Wellcome Sanger Institute Scientific Operations: DNA Pipelines collective are listed here: https://doi.org/10.5281/zenodo.4790455. Members of the Tree of Life Core Informatics collective are listed here: https://doi.org/10.5281/zenodo.5013541. Members of the Darwin Tree of Life Consortium are listed here: https://doi.org/10.5281/zenodo.4783558.
