# Supplementary material for: The genome sequence of the red compost earthworm, Lumbricus rubellus (Hoffmeister, 1843)
Source: Wellcome Open Res. 2023 Aug 18;8:354. [Version 1] doi: 10.12688/wellcomeopenres.19834.1 (PMC11015115; doi:10.12688/wellcomeopenres.19834.1)
Supplement: Peer review history

# Reviewer report: Reviewer response for version 1

**DOI:** 10.21956/wellcomeopenres.21966.r77319

**Published:** 2024-04-12

**Authors:** Yoshiaki Morino

**Affiliations:** 1 Institute of Life and Environmental Sciences, University of Tsukuba, Tsukuba, Japan

This paper reports the genome sequence of the red compost earthworm Lumbricus rubellus. The authors constructed a genome assembly using Pacific Biosciences HiFi long reads, 10X Genomics reads, and Hi-C data. Most of these sequences are mapped to 18 chromosome-level scaffolds. The mitochondrial genome was also assembled. The characteristics of this species, its research history, and the rationale for genome sequencing are clearly described. The quality of the assembly is high enough that I believe this genomic data will be a valuable resource for ecology, evolutionary biology and biotechnology. 

 One minor suggestion for the authors is to introduce that this species (or earthworms in general) are hermaphrodites, possibly related to the lack of sex chromosomes.

Are sufficient details of methods and materials provided to allow replication by others?

Yes

Is the rationale for creating the dataset(s) clearly described?

Yes

Are the datasets clearly presented in a useable and accessible format?

Yes

Are the protocols appropriate and is the work technically sound?

Yes

Reviewer Expertise:

Marine invertebrate, Evo-Devo

I confirm that I have read this submission and believe that I have an appropriate level of expertise to confirm that it is of an acceptable scientific standard.

# Reviewer report: Reviewer response for version 1

**DOI:** 10.21956/wellcomeopenres.21966.r65654

**Published:** 2023-09-14

**Authors:** Sudhakar Sivasubramaniam, Arun Arumugaperumal

**Affiliations:** 1 Department of Biotechnology, Manonmaniam Sundaranar University, Tirunelveli, Tamil Nadu, India; 2 Biotechnology, Rajalakshmi Engineering College, Chennai, Tamilnadu, India

The authors have sequenced the complete genome of *Lumbricus rubellus*, an earthworm species. The genome was not sequenced before, but the mitogenome was sequenced before.

 The authors claim 33,426 coding genes and 13,823 non-coding genes. Since the authors declare it as an invasive species, a phylogenetic relationship study could have established the fact better.

 The authors should clearly mention how the worm was cleaned before DNA extraction.

Are sufficient details of methods and materials provided to allow replication by others?

Partly

Is the rationale for creating the dataset(s) clearly described?

Yes

Are the datasets clearly presented in a useable and accessible format?

Yes

Are the protocols appropriate and is the work technically sound?

Yes

Reviewer Expertise:

Stem Cells, Regeneration, Transcriptomics, Genomics, Earthworm biology

We confirm that we have read this submission and believe that we have an appropriate level of expertise to confirm that it is of an acceptable scientific standard.
